# Development of an Automated Cell-Based Assay for the Detection of the Functional Activity of Saxitoxin

**DOI:** 10.3390/toxins18050206

**Published:** 2026-04-29

**Authors:** Rachel Whiting, Isobel Picken, Grace Howells, A. Christopher Green, Chris Elliott, Graeme C. Clark

**Affiliations:** 1 Dstl-Porton Down, Salisbury SP4 0JQ, UKgmhowells@dstl.gov.uk (G.H.); acgreen@dstl.gov.uk (A.C.G.); 2 Institute for Global Food Security, Queens University, Belfast BT9 5DL, UK; chris.elliott@qub.ac.uk

**Keywords:** saxitoxin, automated patch clamp, voltage-gated sodium channel

## Abstract

Saxitoxin (STX) is one of the most potent natural neurotoxins known and is the only marine toxin to be declared a chemical weapon. In both marine and freshwater systems filter feeding organisms can accumulate saxitoxin and human consumption of toxin-contaminated food can result in paralytic shellfish poisoning. Here we highlight for the first time a human cell-based assay for the detection and neutralisation of STX activity on an automated patch clamp (APC) system. We demonstrate that a human embryonic kidney (HEK) cell line expressing human Nav1.6 can rapidly and sensitively detect the presence of a range of sodium ion channel blockers including STX. The use of neutralising monoclonal antibody GT13-A and/or saxiphilin was found to confer specificity to the assay by being able to dissociate between STX (along with closely related analogues) and tetrodotoxin. Finally, the application of the functional assay for the detection of STX in complex samples was evaluated during an international exercise led by the Organisation for the Prohibition of Chemical Weapons (OPCW). The neutralisation of STX activity in blinded samples enabled the indirect detection of the toxin in the relevant samples and provided an alternative orthogonal technique to corroborate the findings of liquid chromatography–mass spectrometry (LC-MS). Collectively this work demonstrates the significant potential for functional assays in the analysis of samples suspected of being contaminated with STX and related sodium ion channel targeting toxins; complementing traditional direct identification methods such as high-performance liquid chromatography with fluorescence detection (HPLC-FLD), LC-MS or enzyme-linked immunosorbent assay (ELISA).

## 1. Introduction

Saxitoxin (STX) is a potent neurotoxin produced by marine dinoflagellates associated with red tides and freshwater cyanobacteria. In both marine and freshwater systems, filter feeding organisms can accumulate STX. The toxin can then enter the food chain through bioaccumulation in shellfish, fish and birds [1]. Human exposures predominantly arise as a consequence of consumption of toxin-contaminated seafood that can result in paralytic shellfish poisoning. Symptoms can develop within minutes, including tingling of the tongue, numbness of the mouth and, if consumed in large enough quantities, can lead to paralysis, asphyxiation and death [2]. There is no antidote for paralytic shellfish poisoning (PSP), only palliative care that includes the administration of fluids to promote toxin excretion from the body and/or, in severe cases, the provision of respiratory support [3].

STX is one of the most potent natural neurotoxins known, the oral LD_50_ is 5.7 µg/kg and the lethal dose by injection is approximately 0.6 µg/kg [4]. STX is listed as a schedule 1 chemical as part of the Chemical Weapons Convention in part due to the potency of the toxin [5,6]. The lethality of STX is due to its ability to target voltage-gated sodium channels (VGSC), which allow rapid influx of sodium ions across the cell membrane [7]. STX blocks the pore of the VGSC preventing sodium ions entering the cell, inhibiting the initiation and propagation of action potentials, resulting in neuromuscular paralysis and potentially death [6,7,8].

An effective method with which to study the direct interaction of STX with VGSC is using whole-cell patch clamp electrophysiology. Traditional whole-cell patch clamp is an involved low-throughput method. However, the use of automated patch clamp platforms coupled with the availability of cell lines, which have been stably transfected to express human VGSCs [9], enables the consideration of this approach for more routine analysis. The automated patch clamp (APC) also offers the potential of being a rapid and highly sensitive approach for the detection of STX via the functional effects of the toxin on the VGSC [10].

For the past 70 years the mouse bioassay (MBA) has been used as the established regulatory method for detecting PSPs [11]. However, due to ethical implications associated with animal use and the requirement to work to the principles of the three Rs (replacement, reduction, refinement), alternative methods for detecting PSPs are preferred. These include HPLC-FLD which is a validated regulatory method for the direct detection of PSPs [12], LC-MS approaches [13,14], radioligand–receptor binding assay (RBA) [15] and immunological methods such as ELISA [1]. These all provide information that could be used to confirm the presence and/or activity of a toxin in a sample.

**Figure 1:**
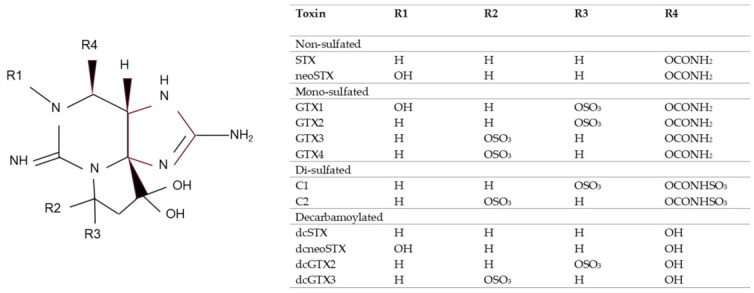
PSP toxin backbone with modifications in the functional side groups (R) of saxitoxin analogues tested in this study. Adapted from McCall et al. [1].

There are 57 known naturally occurring analogues of STX; they share a common structure with modifications in the functional side groups and are commonly grouped according to the variable residues, see Figure 1 [1]. They can be non-sulfated such as STX and neosaxitoxin (neoSTX), mono-sulfated such as the gonyautoxins (GTXs 1–6), or di-sulfated (C1–4 toxins). Decarbamoyl variants of these analogues also exist, including decarbamoyl-saxitoxins (dcSTX, dcneoSTX), decarbamoyl-gonyautoxins (dcGTXs 1–4) and 13-deoxy-decarbamoyl derivatives (doSTX, doGTX2,3) [16].

APC-based approaches when used in combination with recombinant human cell lines expressing VGSCs have the potential to identify a wide range of different STX analogues. Neutralising molecules such as STX-specific antibodies have potential to attribute channel blocking activity to STX and related PSPs. An alternative to neutralising antibodies is Saxiphilin, which is a STX binding protein from the American bullfrog (*Rana catesbeiana*), although it has been found in other amphibians, as well as other organisms including certain fish, arthropods and reptiles [17,18,19,20,21]. Saxiphilin is related to the transferrin class of proteins, it does not bind iron but does strongly bind to STX and so was investigated for its ability to neutralise STX activity in the APC system [22]. Specificity of these neutralising proteins needs to be characterised.

The cells used for this work are a human embryonic kidney (HEK) cell line which expresses human Nav1.6, a VGSC which is localised at nodes of Ranvier, dendrites and synapses [23]. The Nav1.6-HEK cell line was selected for the studies here based on previous research assessing toxicity equivalence factors of PSPs in seven different human Nav subtypes, which showed that the Nav1.6 subunit possessed the strongest equivalence to the MBA. Therefore, the use of cells expressing recombinant human Nav1.6 in conjunction with an APC system offered potential as a functional assay for STX detection and would also support the three Rs principles. A murine APC system has previously been documented for use as a detection tool for the related PSP tetrodotoxin [10]. This work aimed to establish for the first time an optimised protocol using human cells for the detection of an STX related molecule utilising its functional properties towards the relevant human sodium ion channel. The specificity of the technique for different analogues of the toxin—through the use of neutralising proteins—has also been assessed. Our work compliments a recent report demonstrating saxitoxin neutralisation with saxiphilin in an APC system using a different sodium ion channel to the one utilised here [24]. Additionally, we highlight the robustness of the technique through its efficacy at detecting STX within blinded samples provided during an international biotoxin proficiency testing scheme.

## 2. Results

### 2.1. STX and Tetrodotoxin (TTX) Concentration–Response

**Figure 2:**
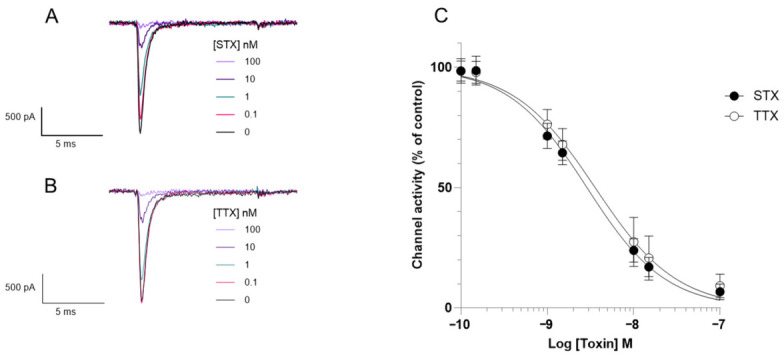
The effect of STX and TTX on Nav1.6-HEK cells. Representative electrophysiological traces at different concentrations of (**A**) STX and (**B**) TTX. (**C**) Concentration–response curves show that increasing concentrations of STX and TTX reduce Nav1.6 currents. Each data point shows the mean ± SD from 19 to 35 cells measured in 4 independent experiments.

Firstly, the effect of STX and TTX on Nav1.6 voltage-dependant sodium currents was measured. Whole-cell APC was used to obtain electrophysiological recordings and to determine the half-maximal inhibitory concentrations (IC_50_). Depolarisation from a holding potential of −90 mV to 0 mV for 10 ms elicits a transient inward current of ~0.5 to ~2 nA in Nav1.6-HEK expressing cells (Figure 2A,B). Increasing concentrations of both STX (Figure 2A) or TTX (Figure 2B) reduce the peak inward current causing almost complete block at 100 nM. These results provide evidence that the Nav1.6-HEK cells are a suitable model for assessing sodium channel blocking agents and the APC an appropriate platform for analysis. STX and TTX elicited a concentration-dependant inhibition of whole-cell current through Nav1.6 channels (Figure 2), with an IC_50_ of 2.9 nM for STX and 3.6 nM for TTX. These similar IC_50_ values demonstrate that whole-cell APC cannot distinguish between STX and TTX on the basis of potency; something else is needed.

### 2.2. Assessment of Potential STX Neutralising Agents

**Figure 3:**
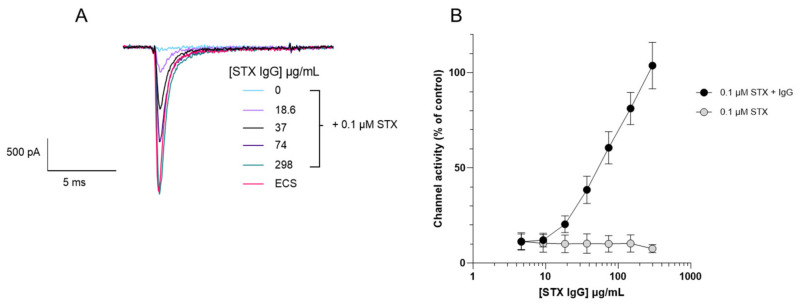
Evaluation of the neutralising properties of monoclonal IgG GT13-A towards saxitoxin through measuring functional activity on Nav1.6-HEK cells. (**A**) Representative electrophysiological traces at different concentrations of monoclonal IgG GT13-A with a fixed concentration of 0.1 µM STX. (**B**) Concentration–response curve shows that increasing concentrations of monoclonal IgG GT13-A neutralises the Nav1.6 channel blocking effect of 0.1 µM STX. For the STX control, without GT13-A IgG, data were acquired at concentrations of vehicle identical to the matched, IgG-treated, groups. These data are plotted against the equivalent IgG concentration. Each data point shows the mean ± SD from 16 to 23 cells measured in 3 independent experiments.

**Figure 4:**
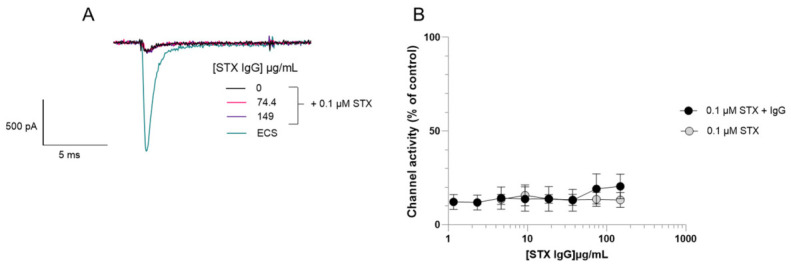
Evaluation of the neutralising properties of polyclonal IgG towards saxitoxin through measuring functional activity on Nav1.6-HEK cells. (**A**) Representative electrophysiological traces at different concentrations of polyclonal STX IgG with a fixed concentration of 0.1 µM STX. (**B**) Concentration–response curve shows that increasing concentrations of STX polyclonal IgG does not neutralise the Nav1.6 channel blocking effect of 0.1 µM STX at concentrations up to 149 µg/mL. For the STX control, without polyclonal IgG, data were acquired at concentrations of vehicle identical to the matched, IgG-treated, groups. These data are plotted against the equivalent IgG concentration. Each data point for 0.1 µM STX + IgG shows the mean ± SD from 18 cells measured in 3 independent experiments. For comparison, the mean ± SD from 3 cells exposed to 0.1 µM STX measured in 1 experiment is shown.

GT13-A, a monoclonal antibody raised to GTX2/3, was evaluated to determine its ability to neutralise the sodium channel blocking effects of STX. Varying concentrations of monoclonal IgG GT13-A were pre-incubated with 0.1 µM STX and the whole-cell current through Nav1.6 channels was measured using whole-cell APC (Figure 3). The monoclonal IgG GT13-A showed a concentration-dependent neutralisation of STX, with the highest concentration (298 µg/mL) completely neutralising 0.1 µM STX, channel activity was not inhibited and was identical to control values. A commercially available STX polyclonal IgG was also assessed but this did not demonstrate any STX neutralising ability in the assay performed (Figure 4).

**Figure 5:**
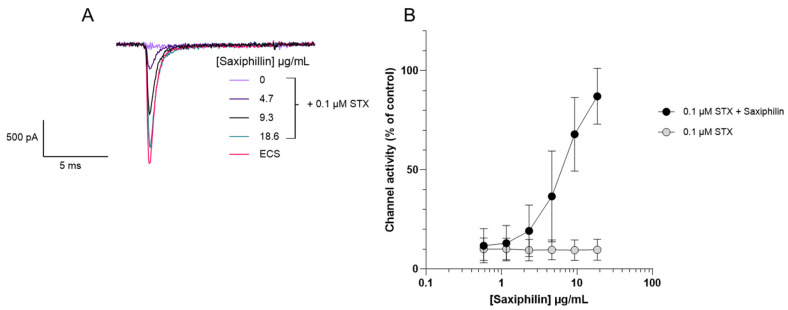
Evaluation of the neutralising properties of saxiphilin towards saxitoxin through measuring functional activity on Nav1.6-HEK cells. (**A**) Representative electrophysiological traces at different concentrations of saxiphilin with a fixed concentration of STX 0.1 µM. (**B**) Concentration–response curve shows that increasing concentrations of saxiphilin neutralises the Nav1.6 channel blocking effect of 0.1 µM STX shown by the increasing channel activity measured with increasing amount of saxiphilin. For the STX control, without saxiphilin, data were acquired at concentrations of vehicle identical to the matched, IgG-treated, groups. These data are plotted against the equivalent IgG concentration. Each data point shows the mean ± SD from 20 to 27 cells measured in 3 independent experiments.

Recombinant saxiphilin was also examined for its ability to neutralise the sodium channel blocking effects of STX. Varying concentrations of saxiphilin were pre-incubated with 0.1 µM STX and whole-cell current through Nav1.6 channels was measured using the APC (Figure 5). Saxiphilin neutralised STX in a concentration-dependent manner, with 87% channel activity restored at the highest concentration of saxiphilin (19 µg/mL).

### 2.3. Detection of the STX Analogues Activity Within the Functional Assay

**Figure 6:**
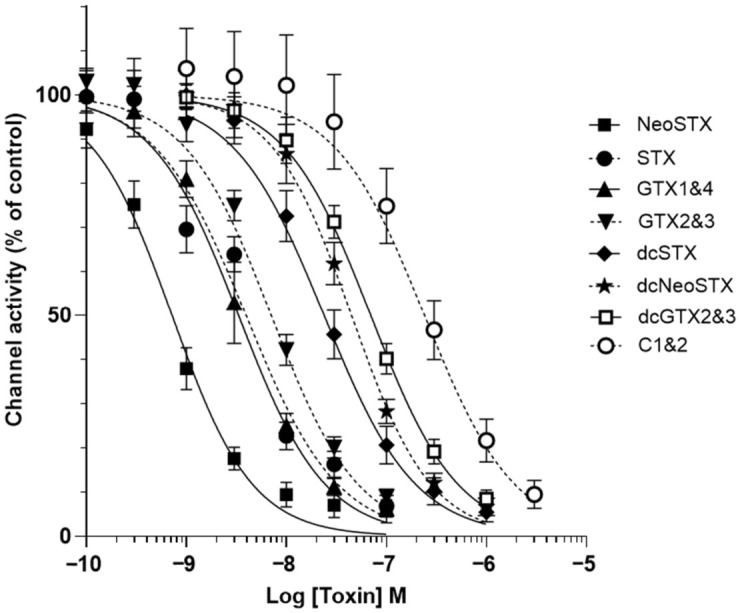
Concentration–response relationships for STX analogues within the functional assay. A concentration-dependent reduction in channel activity was measured through Nav1.6 for all STX analogues tested, neoSTX (IC_50_ 0.7 nM) was the most potent and C1&2 (IC_50_ 239.7 nM) the least. Each data point shows the mean ± SD from 10 to 17 cells measured in 2 independent experiments.

The effects of a range of STX analogues was also assessed within the functional assay. A representative range of commercially available reference standards of STX analogues provided concentration–response data (Figure 6). The non-sulfated analogue neoSTX was found to be the most potent: (IC_50_ 0.7 nM) closely followed by GTX1&4 (IC_50_ 3.6 nM), STX (IC_50_ 3.9 nM) and GTX2&3 (IC_50_ 7.0 nM). The decarbamoylated variants of the analogues were less potent: dcSTX (IC_50_ 27.1 nM), dcneoSTX (IC_50_ 46.9 nM), dcGTX2&3 (IC_50_ 69.6 nM), and the least potent was the di-sulfated C1&2 (IC_50_ 239.7 nM). The concentration of each STX analogue which reduced channel activity to approximately 10% was taken forward to investigate whether the monoclonal IgG GT13-A and/or saxiphilin have neutralising effects on these analogues.

### 2.4. Assessment of Monoclonal IgG GT13-A and Saxiphilin Ability to Neutralise the Activity of STX Analogues and TTX Within the Functional Assay

**Figure 7:**
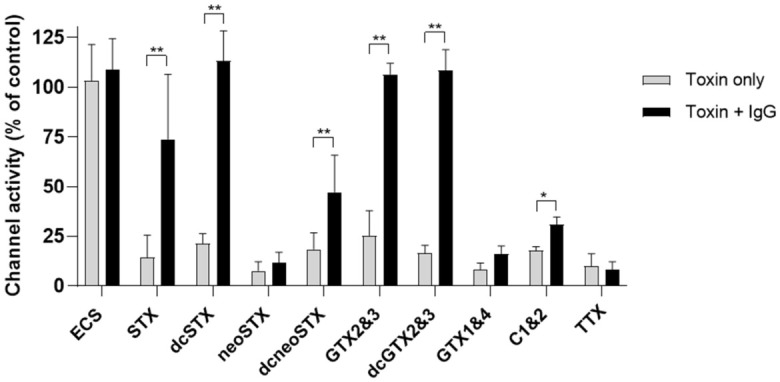
Assessment of the cross-reactivity and neutralising properties of monoclonal IgG GT13-A (anti-saxitoxin antibody) from the effects of STX analogues and TTX within the functional assay; extracellular solution (ECS) was included as a vehicle control. In addition to STX, the monoclonal IgG GT13-A also cross-reacted and neutralised the decarbamoylated analogues (dcSTX and dcGTX2&3) and GTX2&3 but did not neutralise any of the other saxitoxin analogues tested or TTX. Each data point shows the mean ± SD from 10 to 32 cells measured in 2 independent experiments, * *p* < 0.05, ** *p*< 0.01.

**Figure 8:**
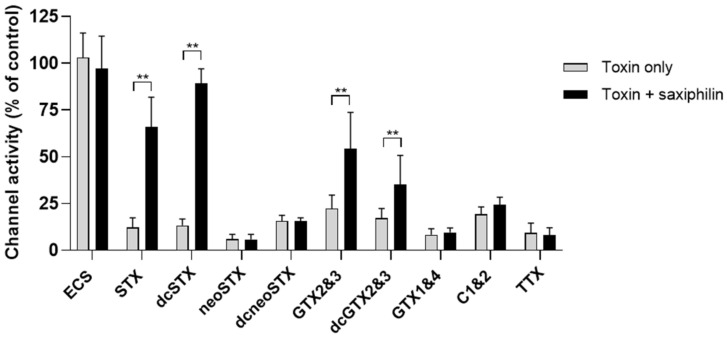
Assessment of the neutralising properties of saxiphilin from the effects of STX analogues and TTX within the functional assay; extracellular solution (ECS) was included as a vehicle control. In addition to STX, the saxiphilin neutralised the decarbamoylated analogue of STX (dcSTX) but did not neutralise any of the other saxitoxin analogues tested or TTX. Each data point shows the mean ± SD from 10 to 36 cells measured in 2 independent experiments, ** *p* < 0.01.

Monoclonal IgG GT13-A (148.8 µg/mL) and saxiphilin (18.6 µg/mL) were incubated with a selection of STX analogues from each class (non-sulfated STX and neoSTX, mono-sulfated GTX2&3, GTX1&4, di-sulfated C1&2 and decarbamoyl versions dcSTX, dcneoSTX, dcGTX2&3) and TTX, a well-characterised sodium channel blocker, to determine cross-reactivity. The anti-saxitoxin monoclonal IgG GT13-A was found to cross react by neutralising GTX2&3 and the decarbamoyl versions of STX and GTX2&3 (Figure 7). In addition to neutralising STX, saxiphilin neutralised decarbamoyl STX (Figure 8). Saxiphilin demonstrated more selectivity than monoclonal IgG GT13-A. No protection was afforded to the Nav1.6-HEK cells by either the monoclonal antibody or saxiphilin from TTX, highlighting the specificity of the functional assay when used in combination with either of these molecules.

### 2.5. Assessment of the Functional Assay to Detect Saxitoxin Within OPCW Biotoxin Samples

**Figure 9:**
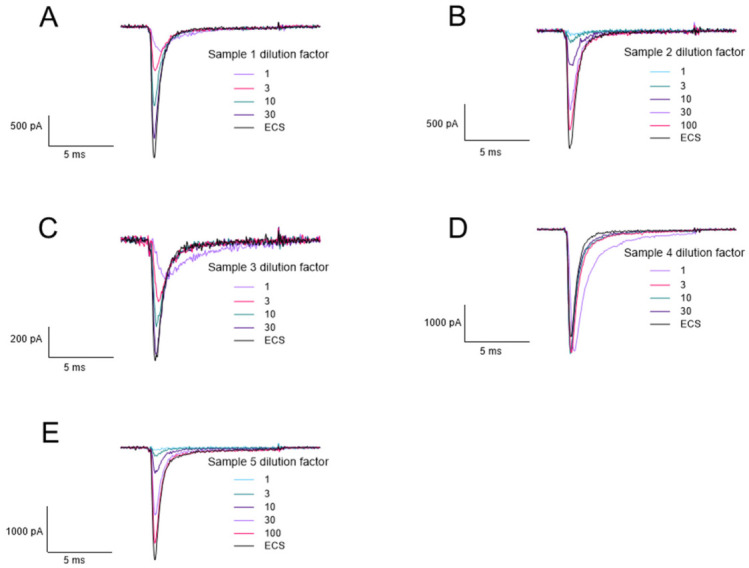

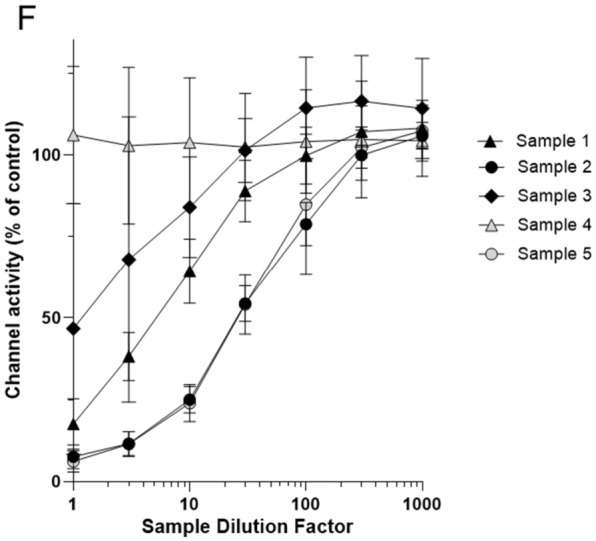
Measurement of the effect of the OPCW biotoxin exercise samples on Nav1.6 channel activity within the functional assay. (**A**–**E**) Representative electrophysiological traces for each sample at a range of dilutions. (**F**) Biotoxin exercise samples concentration–response. Samples 1, 2, 3 and 5 caused a reduction in Nav1.6 channel activity and as each sample was diluted the effect on channel activity decreased. Sample 4 did not reduce Nav1.6 channel activity. Each data point shows the mean ± SD from 6 to 11 cells measured in 2 independent experiments.

The 7th biotoxin exercise for the analysis of samples of biologically derived toxins was organised by the OPCW and focused on STX detection. The OPCW provide the samples which may contain saxitoxin, saxitoxin analogues or no toxin. The sample components were revealed after the exercise completed (Table 1 summarises the spike in the list provided by the OPCW). Our approach included identification using LC-MS (data supplied as Supplementary Information) and APC, which were carried out in parallel. Mass spectrometry was able to directly identify the presence of saxitoxin in samples 1 (see Supplementary Figures S1 and S2) and within samples 3 and 5. Participation in the biotoxin exercise also enabled testing of the functional assay with independantly prepared samples and the determination of active Nav channel blocker in the samples in order to corroborate findings of the direct detection made by LC-MS. The effect of each sample on Nav1.6 sodium currents was measured. The trace data in Figure 9A–C,E clearly shows—as following dilution—that there is a commensurate increase in the current measured through the Nav1.6 channels correctly indicating that a PSP was present within samples 1 (STX only), 2 (neoSTX), 3 (STX only) and 5 (both neoSTX and STX). Figure 9D shows that sample 4 did not reduce the current measured through the Nav1.6 channel, indicating that this sample did not contain saxitoxin (i.e., consistent with the finding of LC-MS). We have observed changes in the activation/inactivation current kinetics in the trace data (Figure 9A–E) which is most prominent at the lowest dilution factor. The samples 1, 2, 3 and 5 (spiked with either STX, neoSTX or both molecules) elicited a concentration-dependant effect on whole-cell current measured through Nav1.6 channels (Figure 9F), demonstrated by the increase in current magnitude as the samples are diluted.

### 2.6. Use of Monoclonal IgG GT13-A to Specifically Identify STX or Related Molecules in Blinded Samples

**Figure 10:**
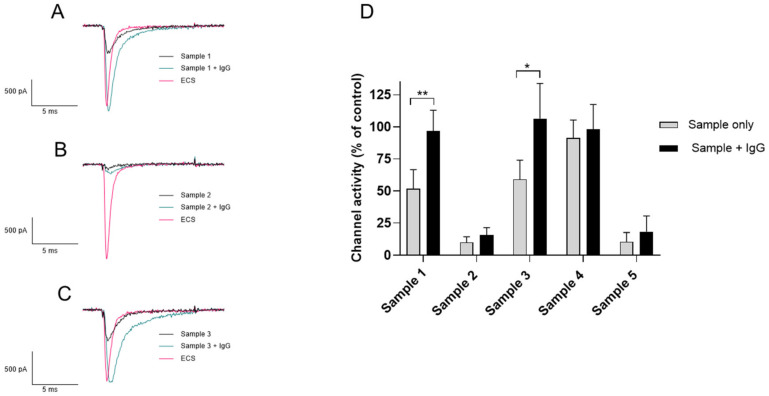
Neutralisation of Nav1.6 channel activity associated with blinded samples provided during the OPCW biotoxin exercise by monoclonal IgG GT13-A. (**A**–**C**) Representative electrophysiological traces for samples 1, 2 and 3, respectively, each trace shows the sample alone and in the presence of monoclonal IgG GT13-A. (**D**) Monoclonal IgG GT13-A neutralised the reduction in Nav1.6 channel activity for samples 1 and 3; however, it showed no neutralisation of samples 2 and 5. Each data point shows the mean ± SD from 6 to 11 cells measured in 2 independent experiments, * *p* < 0.05, ** *p* < 0.01.

The functional assay was able to identify which samples contained compounds which could block the Nav1.6 channel, reducing the whole-cell current measured. The next step was to identify whether the channel blocking compound was specific to STX, GTX2&3 or their decarbamoylated analogues dcSTX and dcGTX2&3. The neutralisation effects of monoclonal IgG GT13-A towards the blinded samples previously identified as having functional activity on Nav1.6-HEK cells was measured (Figure 10). As demonstrated in Figure 10A,C,D, the reduction in Nav1.6 whole-cell current in the presence of samples 1 and 3 was neutralised when monoclonal IgG GT13-A was added. This indicated that STX or a related analogue toxin (dcSTX, GTX2&3 or dcGTX2&3) was the Nav1.6 channel blocker present within these samples. Monoclonal IgG GT13-A did not however neutralise the reduction in Nav1.6 whole-cell current observed with samples 2 and 5 (Figure 10B,D), indicating that the Nav1.6 channel blocker present in these samples was not STX. Orthogonal analysis during the biotoxin exercise using LC-MS analysis confirmed that STX was only present in samples 1 and 3 whereas sample 2 contained neoSTX, sample 5 contained both STX and neoSTX, and sample 4 was a matrix blank. In addition, although a full validation had not taken place for either technique at this juncture, both demonstrated their ability to identify saxitoxin in the nanogram/mL range (i.e., through the use of positive controls and within spiked samples provided for the exercise). A summary of the biotoxin exercise sample composition, the functional activity identified, and the specificity provided through monoclonal GT13-A neutralisation is shown in Table 1. When the sample compositions were released by the OPCW, the results of the APC activity and neutralisation assays were consistent with these. The predicted order of potency for the samples (using data from Figure 9) would be 2 = 5 > 1 > 3 >> (4 inactive); this same order of potency was observed using the functional assay developed in this study (Figure 9).

## 3. Discussion

Current methods for the direct detection of STX include ELISAs [1,25,26], HPLC-FLD [12] or LC-MS [27,28]. However these methods are limited in terms of confirming toxicity in a biological system. Methods which could provide further information about toxicity include the MBA, RBA and APC [10,11,15]. The MBA can provide information on the toxicity of a sample This technique has its disadvantages: the robustness of the assay, strain of the mouse, and pH of the extract have been shown to affect the result. The validity of the method has also been questioned as the MBA uses intraperitoneal (IP) injection, which is not the route of human exposure [29]. The quantity of animals required for testing large numbers of samples has ethical implications, particularly in regard to the 3Rs, and alternative methods which do not involve animals are preferred [30]. The RBA does not require the use of live animals; however, animal brain homogenates still need to be utilised for this method [31]. Therefore, the development of new APC approaches that use cultured cells represent an attractive option in a variety of contexts (i.e., research tool, testing of medical treatments) including the detection of ion channel toxins. The use of Nav1.6-HEK cells in the functional assay allows the assessment of active STX affects towards the human Nav 1.6 (Figure 2). This indirect detection method provides information about toxicity and functionality of the compound in a biological system which would complement other traditional direct identification methods such as LC-MS.

The initial experiments assessing the potency of STX and TTX on Nav1.6 in the functional assay gave an IC_50_ value of 2.9 nM for STX in line with previously reported results in a different APC system [9], traditional patch clamp [32] and a cultured primary neuronal cell assay [33]. The IC_50_ value of 3.6 nM for TTX is comparable to the values reported by Campas et al. [10], which were obtained using an APC system. This demonstrates the suitability of the cells and APC for assessing sodium channel blocking activity.

Confirming that the channel blocking compound was STX required neutralisation of the channel block and a demonstration of an increase in channel activity. Patch clamp electrophysiology has been used to demonstrate the ability of antibodies to neutralise snake [34,35,36,37] and scorpion venom [38] and of saxiphilin to neutralise STX activity at the Nav1.4 from the golden poison frog (*Phyllobates terribilis*) [39]. However, this is the first time that an automated electrophysiology platform and the human Nav1.6 ion channel has been exploited to demonstrate neutralisation of STX and certain analogues by a monoclonal IgG and the STX binding protein, saxiphilin. The monoclonal IgG GT13-A used in the functional assay is a monoclonal antibody raised to GTX2/3, originally developed to be incorporated into an enzyme immunoassay (EIA) for monitoring toxin-contaminated shellfish for GTX components and STX [40]. The results in Figure 3 show that the monoclonal antibody is suitable for use in a functional assay to neutralise the channel blocking actions of STX which is in itself a novel finding. Figure 4 demonstrates the importance of selecting the correct antibody as the STX polyclonal IgG did not neutralise the channel blocking actions of STX.

A representative range of STX analogues were selected to determine the cross-reactivity of the monoclonal IgG GT13-A and saxiphilin. An initial assessment of the potency of each analogue showed the carbamate toxins were the most potent, including neoSTX, STX and GTX 1-4. The decarbamoyl toxins (dcneoSTX, dcSTX, dcGTX 2/3) had intermediate toxicity and the di-sulfated toxin (C1&2) was less potent. This order of toxicity is the same as described by Zakrzewska S [24] and Etheridge SM [3]. Toxicity equivalence factors (TEFs) for STX analogues using data determined by the voluntary feeding, gavage or IP injection of pure toxins to mice supports the results of our STX analogues toxicity assessment [29]. Traditional patch clamp [32] and Neuro2A cell-based assay [41] assessment of STX analogues agree with our data that neoSTX is the most potent and C1&2 the least potent.

The monoclonal IgG GT13-Ademonstrated cross-reactivity and associated neutralising properties within the assay towards dcSTX, GTX 2/3 and dcGTX 2/3 (Figure 7) were expected, as this antibody was raised to GTX 2/3 [40]. Monoclonal antibody IgG GT13-A did not cross react or neutralise the effects of neoSTX, dcneoSTX or GTX1&4 in the functional assay. The same selectivity was also observed when this antibody was used in biosensors [42,43]. These biosensors also bound C1&2 which was not observed through neutralisation of activity in the functional assay; no increase in channel activity was observed with C1&2 in the presence of IgG GT13-A. As this analogue was the least potent, a higher concentration of toxin was added to cause a reduction in channel activity comparable to the other analogues. Therefore, it is probable IgG GT13-A did bind to C1&2, but the fixed concentration of antibody (149 µg/mL) was insufficient to neutralise enough C1&2 to impact its inhibition of channel activity.

Participation in the 7th OPCW biotoxin exercise provided the opportunity to use the functional assays to analyse samples of seawater spiked with STX or STX analogues. A positive and negative control, as well as three unknown samples, were provided by the OPCW. There has been a drive towards confirming the activity of a toxin which is present in a sample. Sample analysis was carried out blind and the results in Figure 9 demonstrated the presence of a Nav channel blocking compound in samples 1, 2, 3 and 5, with sample 4 containing no Nav channel blocking compound (this was eventually identified as the negative control). In the trace data in Figure 9A–E, we observe changes in the activation/inactivation current kinetics which is most noticeable at the lowest dilution factor. This is likely to be an effect of the seawater matrix changing the ion concentrations, as this was observed in sample 4 (Figure 9D) which contained seawater only. As the samples are diluted in buffer, the change in activation/inactivation current kinetics is no longer observed, supporting this conclusion. Overall, the functional assay confirmed the presence of active toxin in the correct samples. Moreover, once the composition of the samples was known the order of potency of these samples when assessed by serial dilution (Figure 9F) and matched that predicted from the known potencies of the toxins present (Figure 6).

The use of the functional assay in combination with the neutralisation properties of monoclonal antibody IgG GT13-A (Figure 10) indicated that samples 1 and 3 are likely to contain STX, dcSTX, GTX2&3 or dcGTX2&3, as the reduction in channel activity observed when only the sample was present was prevented when monoclonal IgG GT13-A was added to the sample with channel activity returning to control levels. There was no neutralisation of activity observed for samples 2 and 5, demonstrating that these samples contain VGSC blockers that are not neutralised by the antibody, so may, for example, contain toxins such as neoSTX or TTX (Figure 7). When the spiking list was released by the OPCW it confirmed that samples 2 and 5 contained neoSTX. However, sample 5 contained STX and neoSTX, so the neutralisation assay did not demonstrate any increase in channel activity of the sample in the presence of monoclonal IgG GT13-A compared to the sample alone. Looking at the spiking list concentrations it became clear why we did not observe any neutralisation, as sample 5 contained 20 ng/mL STX and 50 ng/mL neoSTX. As we have demonstrated in Figure 6, neoSTX is more potent than STX and was present in the sample at a higher concentration, even though the monoclonal IgG GT13-A neutralised the STX, this was masked by the activity of the more potent neoSTX. This is demonstrated in Figure 9F, as samples 2 and 5 have almost identical channel activity as the sample is diluted, even though sample 5 has 20 ng/mL STX present in addition to the 50 ng/mL neoSTX present in sample 2.

## 4. Conclusions

The automated functional assay developed here provides a new high throughput human cell-based method for indirect identification of the presence of STX, STX analogues and TTX in samples. This is achieved by measuring the effects of the toxin upon continuous cell line expressing human Nav1.6 ion channel. The approach was found to be an effective method for detecting the activity of a range of PSP toxins including during an international exercise. In addition, we have demonstrated that the monoclonal IgG GT13-A and saxiphilin can confer specificity to the assay by neutralising the channel blocking effects of STX and certain analogues and not TTX. Finally, the monoclonal IgG GT13-A neutralisation assay demonstrated the presence of active toxins in samples from the 7th OPCW biotoxin exercise. This new indirect approach provided an orthogonal functional-based technique that complemented the direct toxin identification made by LC-MS, enabling the samples containing STX toxin to be correctly identified. These results demonstrate the potential applicability of this human Nav1.6-HEK cell-based functional assay in supporting the analysis of samples for the presence of STX and related sodium ion channel targeting toxins

## 5. Materials and Methods

### 5.1. Materials

STX dihydrochloride and STX analogues were obtained from National Research Council (Halifax, NS, Canada). TTX standard was purchased from Tocris Bioscience (Bristol, UK). The APC QPatch II and 16 channel Qplates were supplied by Sophion Bioscience (Ballerup, Denmark). Electrophysiological experiments on QPatch II utilised extracellular solution (ECS) which contained NaCl 145, KCL 4, MgCl_2_ 1, CaCl_2_ 2, HEPES 10 and glucose 10 in mM, at pH7.4. Intracellular solution (ICS) contained CsF 140, EGTA 1, HEPES 10, CsOH 5 and NaCl 10 in mM, at pH 7.25. Type 1 ultrapure water (18.2 MΩ/cm) was used to prepare the solutions, supplied by Avidity Science (Aylesbury, UK). ECS was used to make working concentrations of toxins and dilute biotoxin exercise samples (initial dilution of 163 µL biotoxin exercise sample + 837 µL ECS referred to as dilution factor 1). HEK293 expressing human Nav1.6 were purchased from SB Drug Discovery (Glasgow, UK). Monoclonal IgG GT13-A was supplied by Queens University Belfast (Northern Ireland, UK). STX polyclonal IgG was purchased from Agrisera (Vannus, Sweden). Recombinant saxiphilin protein was purchased from Abcam Ltd. (Cambridge, UK). All other chemicals were purchased from Sigma-Aldrich Ltd. (Gillingham, UK).

### 5.2. Nav1.6-HEK Cell Line Maintenance

Cells were maintained in minimum essential medium (MEM) with heat inactivated foetal bovine serum (FBS) (10%), L-glutamine (2 mM), penicillin/streptomycin (50 units/mL), blasticidin (2 µg/mL) and geneticin 418 (0.6 mg/mL) in an incubator maintained at 37 °C in a 5% CO_2_ humid atmosphere. Cells were grown to 70/80% confluence and passaged every 3–4 days using TrypLE express. For electrophysiology experiments the cells were washed with phosphate-buffered saline (PBS) dissociated with TrypLE express then resuspended in Ex-Cell ACF CHO medium (3.5 × 10^6^ cells/mL) and allowed to recover with stirring on QPatch II for a minimum of 45 min before use in an experiment.

### 5.3. Automated Patch Clamp Recording—Assessing the Ability of STX, STX Analogues and Biotoxin Exercise Samples to Block the Whole-Cell Current Measured Through Nav1.6 Channels

The electrophysiological changes on Nav1.6-HEK cells were recorded using QPatch II 16, which allows analysis of up to 16 individual cells simultaneously. A holding potential of −90 mV was utilised, and the Nav1.6 channels were activated by a depolarizing step to 0 mV for 10 ms for all experiments. Current recordings were sampled at 25,000 Hz and filtered in software with a 5000 Hz cut-off (Bessel). A leak protocol 3 10 ms hyperpolarizing pulses to −120 mV was used to measure leak current for the application of leak subtraction. An initial Nav 1.6 current of 500 pA and seal resistance of 100 MΩ defined acceptable recordings. The STX, STX analogues or biotoxin exercise samples were added to the patched cells in a cumulative fashion and for each cell, TTX (1 µM) was applied after the compound applications of the toxins or ECS only control to act as a positive control as Nav1.6 are TTX-sensitive.

### 5.4. Automated Patch Clamp Recording—Assessment of Potential STX Neutralising Agents

Fixed concentrations of STX (0.1 µM), TTX (0.1 µM) or ECS control had varying concentrations of STX IgGs, saxiphilin or ECS control added and were incubated at room temperature for at least 1 h before application to the patched cells. The Nav1.6 channels were activated on QPatch II, as described Section 5.3, and the potential neutralising agents (monoclonal IgG GT13-A 4.7–298 µg/mL, polyclonal IgG 4.7–149 µg/mL and saxiphilin 0.6–18.6 µg/mL) with toxin or ECS control were added sequentially to the patched cells starting with the sample containing the highest concentration of the neutralising agent.

### 5.5. Automated Patch Clamp Recording—Neutralisation Assay

The neutralising properties of the monoclonal IgG GT13-A and saxiphilin was tested by examining their ability to protect Nav1.6-HEK cells from various STX analogues and TTX. The concentration which reduced channel activity to approximately 10% was selected as the challenge concentration for each STX analogue and TTX. Each toxin was incubated with final concentrations of 149 µg/mL monoclonal IgG GT13-A or 18.6 µg/mL saxiphilin or ECS control, for a minimum of 1 h before application to the patched cells. Analysis of the biotoxin exercise samples was conducted blind, the concentration of toxin in the samples was unknown; therefore, the dilution of the sample which reduced channel activity to approximately 10% was incubated with final concentrations of 149 µg/mL monoclonal IgG GT13-A or ECS control for 1 h before application to the patched cells.

### 5.6. Statistical Analyses

The maximum inward Nav current data was exported from the APC and normalised to saline. All concentration–response data was analysed by performing a Sigmoidal dose–response (variable slope) $Y=Bottom+\;\frac{Top-Bottom}{1+10^{\left(LogIC50-X\right)HillSlope}}$. A 4-parameter logistic fit was used with the bottom of the curve constrained to 0 and the top of each curve constrained to 100. This fit was used to obtain the half-maximal inhibitory concentration (IC_50_).

Differences between toxin/sample alone and in the presence of the potential neutralising agent were assessed using the Kruskal–Wallis test (non-parametric equivalent of the analysis of variance (ANOVA) test), as the data was not normally distributed (according to the Shapiro–Wilk test). Differences were considered statistically significant for *p*-value < 0.05 * or <0.01 **. Statistical analyses were performed using the statistical software GraphPad Prism v10 (San Diego, CA, USA).

### 5.7. LC-MS Analysis of Biotoxin Exercise Samples

Samples were analysed using a Thermo Scientific Vanquish UPLC coupled to Thermo Scientific Q Exactive Plus OrbitrpMS supplied by Thermo Scientific (Loughborough, UK). The instrument method parameters used are referenced in Tables 1 and 3 of information published by the OPCW, New Methods for the Detection, and confirmation of the detection of Saxitoxin in Environmental Samples [44]. Data were acquired using the parallel reaction monitoring (PRM) mode. Criteria stated according to the OPCW reporting criteria was met [45]. The mass accuracy was ≤5 ppm. The ion ratios, for *m*/*z* 282.13091 and *m*/*z* 204.08799, were below the maximum variation allowed for the reference chemical and sample.

This method was used for qualitative analysis only. This method was not validated at time of analysis.

## Figures and Tables

**Table 1:** Summary of functional assay results in assessing the potential presence of saxitoxin within samples provided during the 7th OPCW biotoxin exercise. The biotoxin exercise samples were analysed blind. When information on the spiking concentrations were released it demonstrated that the functional assay, in combination with the use of the neutralising monoclonal antibody GT13-A, could successfully identify STX and related analogues.

Sample Number	Indicative Sample Composition	Actual Sample Composition (ng/mL)	Is the Functional Assay Output Consistent with Sample Identification and Activity?
1	STX, dcSTX, GTX2&3 dcGTX2&3	STX (50)	Yes
2	Active blocker but not neutralised	neoSTX (50)	Yes
3	STX, dcSTX, GTX2&3 dcGTX2&3	STX (30)	Yes
4	No active blocker	Matrix only	Yes
5	Active blocker but not neutralised	STX (20), neoSTX (50)	Yes

## Data Availability

The original contributions presented in this study are included in the article/Supplementary Material. Further inquiries can be directed to the corresponding authors.

## Supplementary Materials

The following supporting information can be downloaded at: https://www.mdpi.com/article/10.3390/toxins18050206/s1, Figure S1: Extracted ion chromatograms (*m*/*z* 282.13091 ± 5 ppm) of saxitoxin. (A) Sea water matrix blank. (B) Sample 1. (C) Standard of Saxitoxin, 60 ng∙mL^−1^ spiked into sea water matrix.; Figure S2: Extracted ion chromatograms (*m*/*z* 204.08799 ± 5 ppm) of saxitoxin. (A) Sea water matrix matched blank. (B) Sample 1. (C) Standard of Saxitoxin, 60 ng∙mL^−1^ spiked into sea water matrix.

## Crown Copyright (2026)

Dstl. This information is licensed under the Open Government Licence v3.0. To view this licence, visit https://www.nationalarchives.gov.uk/doc/open-government-licence/. Where we have identified any third party copyright information you will need to obtain permission from the copyright holders concerned. Any enquiries regarding this publication should be sent to: centralenquiries@dstl.gov.uk.

## Abbreviations

The following abbreviations are used in this manuscript:

APC	Automated patch clamp
C1&2	N-sulfocarbamoyl analogues of STX
Dc	Decarbamoyl
Do	13-deoxy-decarbamoyl analogues of STX
ECS	Extracellular solution
ELISA	Enzyme-linked immunosorbent assay
FBS	Foetal bovine serum
GTX	Gonyautoxin
HPLC-FLD	High-performance liquid chromatography with fluorescence detection
HEK	Human embryonic kidney
IC_50_	Half-maximal inhibitory concentration
ICS	Intracellular solution
IP	Intraperitoneal
LC-MS	Liquid chromatography–mass spectrometry
Nav1.6	Sodium channel Nav1.6
MBA	Mouse bioassay
MEM	Minimum essential medium
NeoSTX	Neosaxitoxin
OPCW	Organisation for the Prohibition of Chemical Weapons
PRM	Parallel reaction monitoring
PSP	Paralytic shellfish poisoning
RBA	Radio-ligand receptor binding assay
SD	Standard deviation
STX	STX dihydrochloride
TEFs	Toxicity equivalence factors
TTX	Tetrodotoxin
VGSC	Voltage-gated sodium channels
