# Supplementary material for: Development of an Automated Cell-Based Assay for the Detection of the Functional Activity of Saxitoxin
Source: Toxins (Basel). 2026 Apr 29;18(5):206. doi: 10.3390/toxins18050206 (PMC13211387; doi:10.3390/toxins18050206)
Supplement: Supplementary file 1 [file toxins-18-00206-s001.zip › toxins-4176998-supplementary.pdf]

Supplementary Materials: Development of an Automated Cell-Based Assay for the Detection of the Functional Activity of Saxitoxin

Rachel Whiting, Isobel Picken , Grace Howells, A. Christopher Green, Chris Elliott and Graeme C. Clark

LC-MS/HRMS data

A

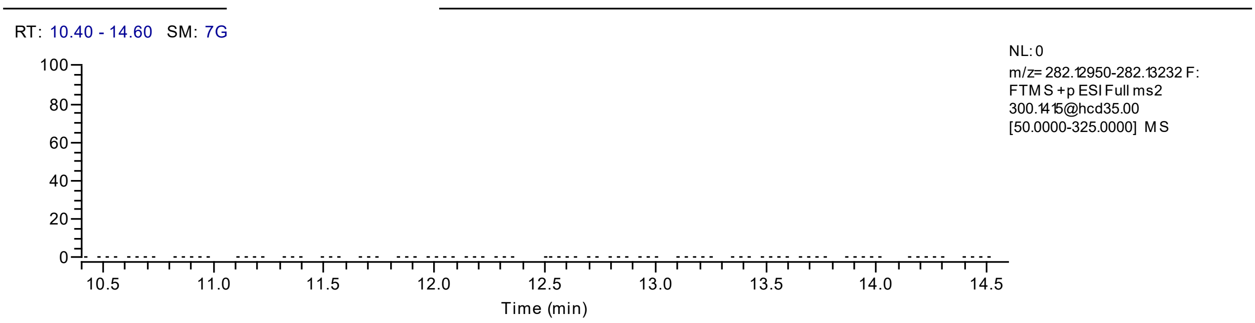

B

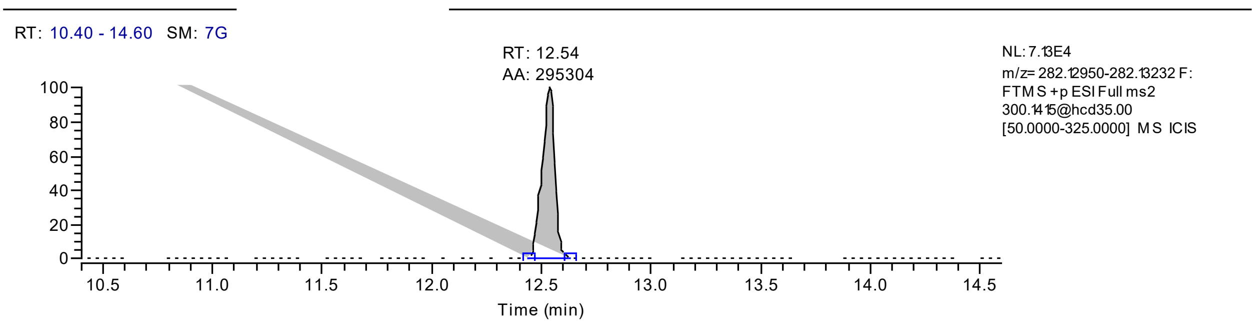

C

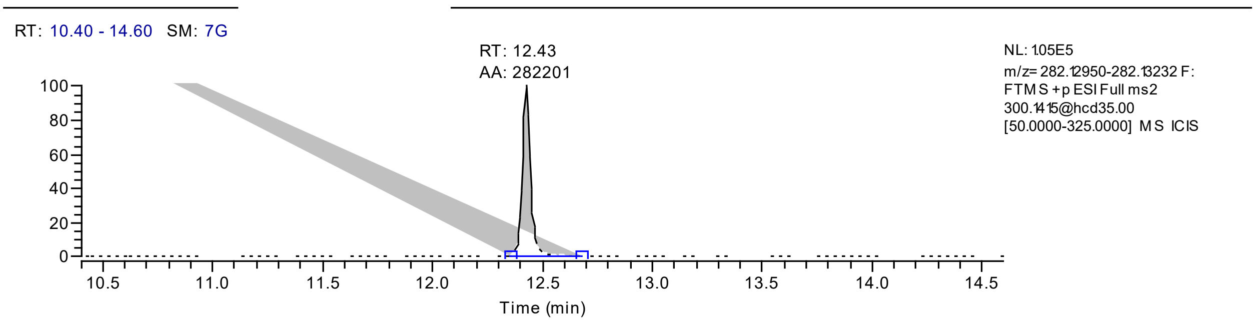

Figure S1. Extracted ion chromatograms (*m*/*z* 282.13091 ± 5ppm) of saxitoxin. (A) Sea water matrix blank. (B) Sample 1. (C) Standard of Saxitoxin, 60 ng·mL^{-1} spiked into sea water matrix.

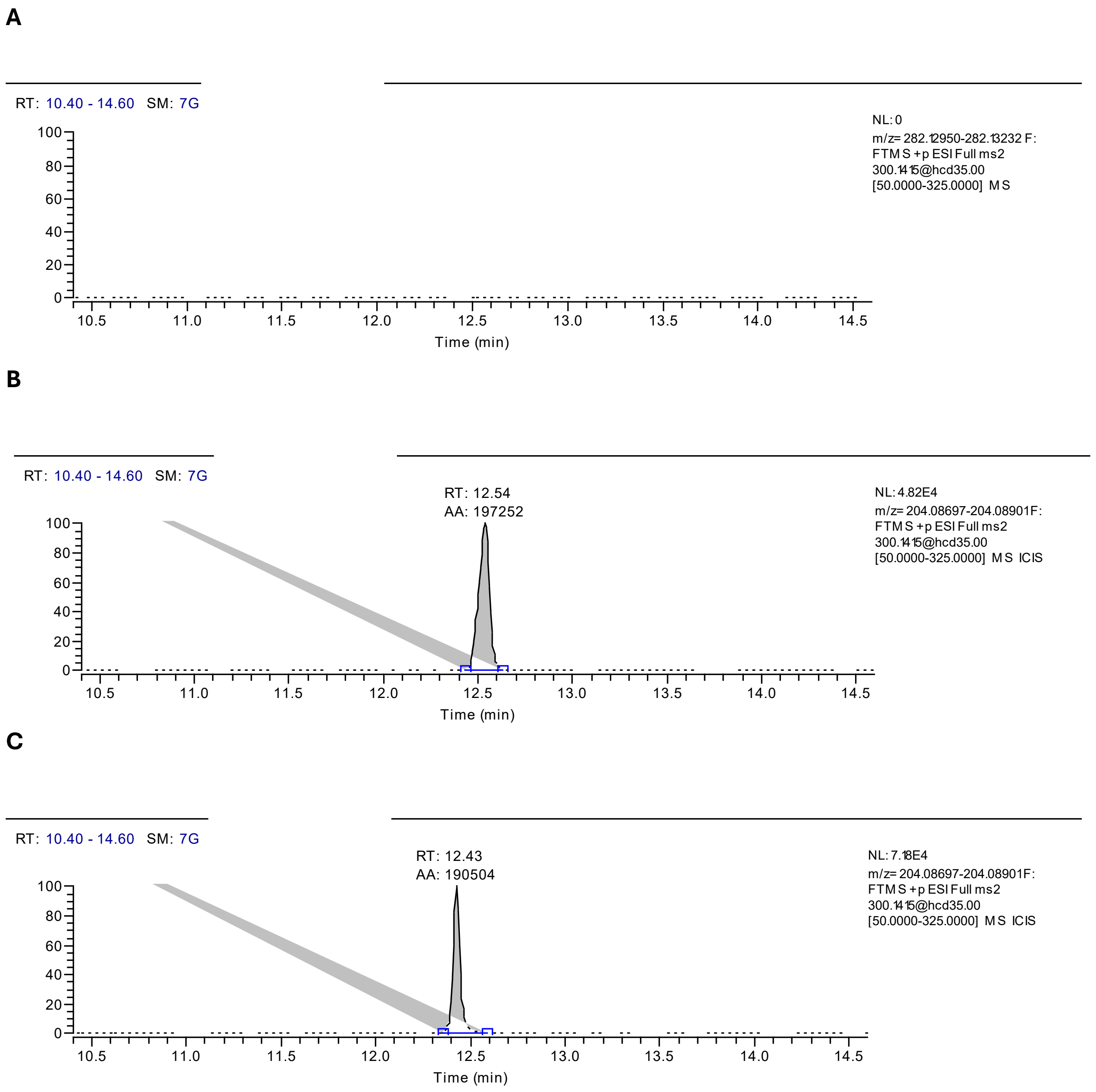

Figure S2. Extracted ion chromatograms (*m/z* 204.08799 ± 5ppm) of saxitoxin. (A) Sea water matrix matched blank. (B) Sample 1. (C) Standard of Saxitoxin, 60 ng·mL^{-1} spiked into sea water matrix.

Samples were analysed using a Thermo Scientific Vanquish UPLC coupled to Thermo Scientific Q Exactive Plus Orbitrap MS. The instrument method parameters used are referenced in table 1 and 3 of information published by the OPCW, New Methods for the Detection, and Confirmation of the Detection of Saxitoxin in Environmental Samples [1]. Data was acquired using parallel reaction monitoring (PRM) mode. Criteria stated according to the OPCW reporting criteria was met [2]. The mass accuracy was ≤ 5 ppm. The ion ratios, for *m/z* 282.13091 and *m/z* 204.08799, were below the maximum variation allowed for the reference chemical and sample.

This method was used for qualitative analysis only. This method was not validated at time of analysis.

References

1. Organisation for the Prohibition of Chemical Weapons. *New Methods for the Detection, and Confirmation of the Detection of Saxitoxin in Environmental Samples*; OPCW: Hague, The Netherlands, 2021.
2. Organisation for the Prohibition of Chemical Weapons. *Guidelines for the Seventh Biotoxin Analysis Exercise*; OPCW: Hague, The Netherlands, 2022.
